# A case report and literature review of rectal lipoma

**DOI:** 10.1097/MD.0000000000034429

**Published:** 2023-10-27

**Authors:** Wenguang Gao, Kun Zhang, Feng You

**Affiliations:** a Shandong University of Traditional Chinese Medicine, Linyi City, Shandong Province, China; b Linyi Traditional Chinese Medicine Hospital, Linyi City, Shandong Province, China; c Department of Radiology, Affiliated Hangzhou First People’s Hospital, Zhejiang University School of Medicine, Hangzhou, ZheJiang, China.

**Keywords:** anal lump, giant rectal lipoma, transanal mass resection

## Abstract

**Rationale:**

Colonic lipomas are uncommon benign submucosal adipose tumors that are usually asymptomatic. In principle, large lipomas can cause symptoms that require further treatment. Here, we report a case of prolapsed giant rectal lipoma and transanal mass resection.

**Patient concerns:**

A 65-year-old male developed rectal mass prolapse with bloody stool for 1 day.

**Diagnoses:**

The pathological findings were rectal lipoma.

**Intervention:**

After resection of the anal tumor, the patient postoperative symptoms quickly disappeared.

**Outcomes:**

No recurrence of the condition was observed after 6 months of follow-up after surgery.

**Lessons:**

It is safe and feasible for us to perform transanal mass resection for giant rectal lipomas that protrude outside the anus.

## 1. Introduction

Lipoma is a benign soft tissue tumor composed of mature adipocytes.^[1]^ It is common on the surface of the body and can also be seen inside the body. Most lipomas have a soft texture, good mobility, and are generally painless.^[2]^ Colorectal lipoma is relatively rare in clinic. It is not easy to find in the early stage. When the tumor is large, abdominal pain, diarrhea and bloody stool may appear. Because of the low growth position, rectal or sigmoid colon lipoma may appear sagging and prolapse out of the anus. The tumor mucosa may have congestion and edema, focal erosion, or even ulceration leading to bloody stool. The clinical manifestations of this disease lack specificity, and some physicians lack understanding and experience, making it prone to misdiagnosis.^[3]^ This article reports a case of large rectal lipoma based on existing literature materials, in order to provide reference for the diagnosis and treatment of colorectal lipoma in clinical practice.

## 2. Case presentation

**Figure 1.**
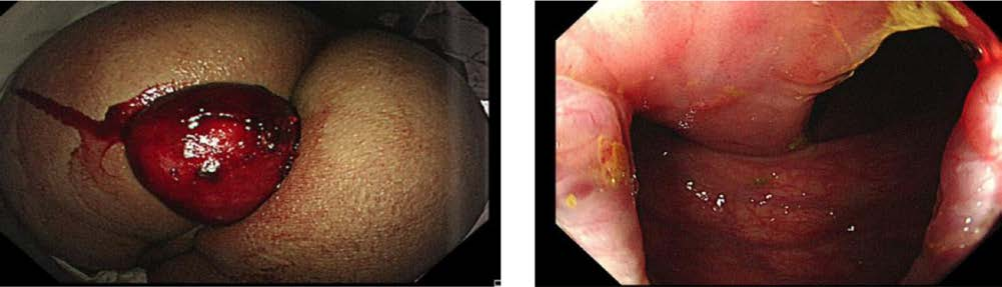
A huge and extraanal lipoma with a long pedicle.

The patient was a 65-year-old male who complained of rectal mass prolapse with blood in the stool for 1 day. Through physical examination, a huge round mass prolapsed from the anus, and no abnormality was found in laboratory examination. Colonoscopy revealed a polypoid mass with a diameter of about 6cm, and it was found that the mass had a long pedicel when it came to the junction of rectum and sigmoid colon (Fig.1). The mass texture is soft and submucosal fat-like tissue was visible when touched by a biopsy forcep. The preoperative diagnosis was colonic lipoma depending on the colonoscopy finding.

**Figure 2.**
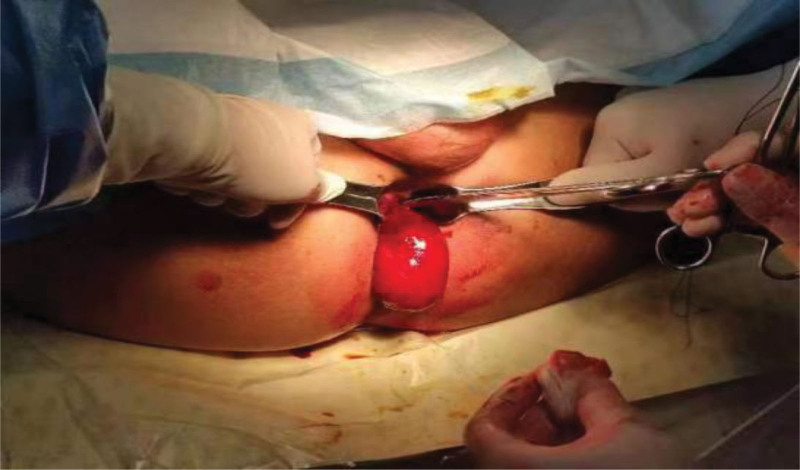
Ligate the tumor with a thread during surgery and remove it.

**Figure 3.**
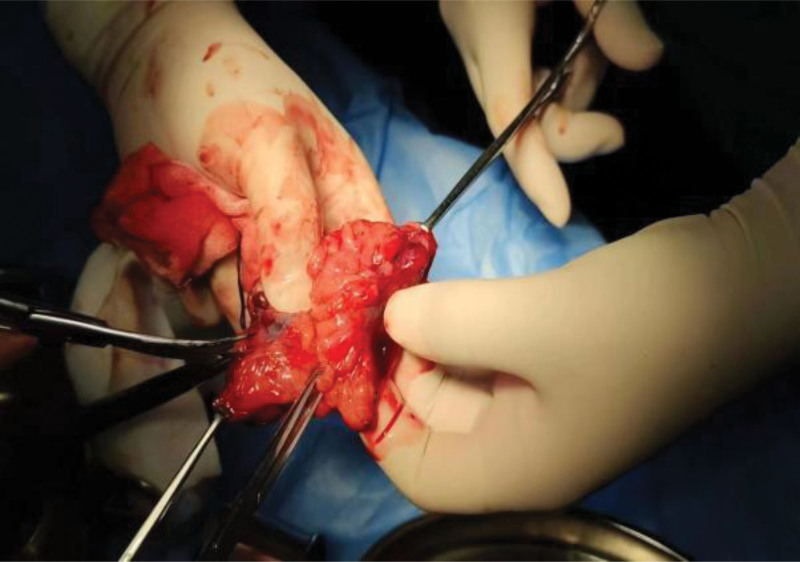
Yellow adipose tissue seen in anatomical specimens.

Due to the large volume of the lump, endoscopic polypectomy cannot be performed, and since the lump has already protruded from the anus, we have decided to perform transanal mass resection for it (Fig.2). Postoperative resection of pathological specimens is shown in Figure 3. The patient was discharged on the 5th day after surgery without any complications. Follow up for 6 months showed no complications.

## 3. Discussion

Colorectal lipoma is a rare intestinal benign tumor in clinic, with a low incidence rate. Rectal lipoma is even rarer, accounting for 3.4% of all colorectal lipomas in incidence rate.^[4]^ The etiology and pathogenesis of colorectal lipoma have not yet been elucidated, and some literature suggests that it may be related to systemic fat metabolism, intestinal fat metabolism disorders, and intestinal malnutrition.^[5]^ The clinical symptoms of colorectal lipoma are related to the size, location, and morphology of the tumor. When the diameter of the tumor is <2 cm, there are often no obvious symptoms, and they are often discovered by chance during physical examinations, autopsies, or other surgeries; When the diameter of the tumor body is ≥2 cm, symptoms such as abdominal pain, diarrhea, and constipation can occur; When the diameter of the tumor body is >3 cm, symptoms such as abdominal lumps, mucus and bloody stools, and anemia may occur.^[6]^ The rectal lipoma in this case occurred at a lower position, at the junction of the rectum and sigmoid colon. The early symptoms were not obvious and could not be found in time. When the tumor grew to a certain extent and prolapsed out of the anus, the patient had foreign body sensation in the anus and pain in the lower abdomen, and because of congestion and edema of the surface mucosa of the tumor, the patient had bloody stool and other symptoms.

The clinical manifestations of colorectal lipoma are nonspecific, and its diagnosis mainly relies on colonoscopy and imaging examination, with the final diagnosis relying on pathological examination.^[3]^ Colonoscopy is an important means of diagnosing colorectal lipomas. Most colorectal lipomas appear as pink masses with intact capsule under colonoscopy, which can have a pedicle, a pedicle, or an unstedicle. The surface is smooth, the texture is soft, and occasionally blood vessels pass through. When using biopsy forceps, one can see: When using biopsy forceps, one can see randomly restored localized pressure marks, known as the “cushion sign”; Use biopsy forceps to lift the mucosa on the surface of the tumor, causing the internal tumor tissue to protrude, known as the “tent effect”; When the mucosa on the surface of the tumor is repeatedly biopsied, it can be seen that fat is exposed through the mucosal tissue, known as the “naked fat sign.”^[7,8]^ If congestion, ulcer and erosion appear on the surface or base of the mass, it is difficult to differentiate it from mass type colorectal cancer at this time. Therefore, some literature points out that repeated biopsy or endoscopic ultrasonography can be used to assist diagnosis when necessary. Colorectal lipoma under endoscopic ultrasonography is characterized by uniform hyperechoic mass in the submucosa.^[9]^ Computed tomography (CT) is more effective in determining the location and quality of colorectal lipomas. The typical manifestations of colorectal lipomas on CT are uniform low-density masses, clear boundaries, no enhancement on enhanced scans, and significant negative CT values (fat density). At the same time, CT can detect coexisting intussusception and clearly embed relevant tissue components.^[10]^ Colorectal lipoma is a non-epithelial benign tumor derived from interstitial tissue. Currently, many mathematicians classify it into 4 pathological types: submucosal type, subserosal type, intramuscular type, and mixed type. Among them, submucosal type is the most important pathological type.^[11]^ The pathological diagnosis of this case is a submucosal fibrolipoma of the rectum, with a diameter of 6 cm, which is rare.

Some literature points out that there are currently 3 main treatment methods for colorectal lipomas, including colonoscopy, laparoscopy, and robotics. Traditional open surgery is less commonly used, and low rectal lipomas closer to the anus can be treated with local resection through the anus.^[12]^ The specific surgical method should be selected based on the size, location, characteristics of the base of the lipoma, and the level of accumulated intestinal wall.^[13]^ Endoscopic resection mainly includes endoscopic submucosal dissection, snare removal, nylon assisted resection, and fat removal. Endoscopic submucosal dissection is mainly suitable for small volume submucosal lipomas. Large volume submucosal lipomas are generally removed using a snare, while for those with a wide base that is difficult to be completely removed at once, nylon ferrule assisted resection or fat removal surgery can be used.^[14]^ Although there is rapid development in endoscopic technology at present, laparoscopic surgery is still recommended for subserosal or intramuscular lipomas.^[15]^ The application of laparoscopic technology can complete tumor resection without damaging the intestinal wall. If the lipoma is large and has a long pedicle, it can be located under the guidance of colonoscopy first, and then laparoscopic incision of the seromuscular layer of the intestinal wall can be performed to peel off the tumor, If the tumor is difficult to remove, a small amount of intestinal canal can be removed. This case belongs to a low position rectal lipoma, which is large and has protruded from the anus. After sacral anesthesia, it is clearly exposed and has no adhesion to the surrounding tissues. To ensure the integrity of the tumor resection, transanal rectal tumor resection is adopted. There is less bleeding during the operation, and the patient returns to the ward safely. The patient condition is stable and there are no complications after the surgery. The patient is discharged 5 days after the surgery. Postoperative follow-up should be strengthened, and regular colonoscopy should be conducted to detect recurrence in a timely manner.

## 4. Conclusion

Colorectal lipoma is a rare disease in the field of anorectal surgery, commonly seen in middle-aged and elderly people, with a certain risk of malignancy. Its clinical symptoms are nonspecific, and colonoscopy and CT examination are important diagnostic methods for this disease. Diagnosed colorectal lipomas should follow the principles of benign tumor treatment, and simple tumor resection should be chosen to minimize damage to the intestinal wall. The surgical method is mainly through endoscopic or surgical resection, and low rectal lipomas can also be removed through the anus. For those who cannot be diagnosed before surgery, rapid intraoperative frozen pathological examination is of great guiding significance for the selection of surgical methods. Regular follow-up colonoscopy should be performed after surgery to detect early recurrence or malignancy. This article demonstrates that for a lipoma that has already protruded outside the anus, transanal mass resection is necessary, and the postoperative lesion can be self-eliminated.

## Acknowledgements

We would like to thank the researchers and study participants for their contributions.

## Author contributions

**Validation:** Kun Zhang.

**Supervision:** Feng You

**Writing – original draft:** Gao Wenguang.

**Writing – review & editing:** Kun Zhang, Feng You.
